# Prediction of recurrence-related factors for patients with early-stage cervical cancer following radical hysterectomy and adjuvant radiotherapy

**DOI:** 10.1186/s12905-023-02853-8

**Published:** 2024-01-31

**Authors:** Gui-Fen Ma, Gen-Lai Lin, Si-Tong Wang, Ya-Yu Huang, Chun-Li Xiao, Jing Sun, Ting-Yan Shi, Li-Bing Xiang

**Affiliations:** 1 Department of Radiation Oncology, Zhongshan Hospital, Fudan University, Shanghai, 200032 China; 2 Department of Radiation Oncology, Zhongshan Hospital, Fudan University, Xiamen Branch, Fujian, 361000 China; 3 Department of Gynecological Oncology, Zhongshan Hospital, Fudan University, Shanghai, 200032 China

**Keywords:** Early-stage cervical cancer, Radical hysterectomy, Adjuvant radiotherapy, Recurrence-free survival, Recurrence rate

## Abstract

**Objective:**

To analyze recurrent factors in patients with clinical early-stage cervical cancer (ESCC) following hysterectomy and adjuvant radiotherapy.

**Methods:**

We collected data from patients with ESCC, staged according to the 2009 Federation International of Gynecology and Obstetrics (FIGO) staging criteria, who underwent hysterectomy followed by adjuvant radiotherapy between 2012 and 2019. These patients were subsequently restaged using the 2018 FIGO criteria. Univariable and multivariable analyses, along with nomogram analyses, were conducted to explore factors associated with recurrence-free survival (RFS).

**Results:**

A total of 310 patients met the inclusion criteria, with a median follow-up time of 46 months. Among them, 126 patients with ESCC were restaged to stage III C1 or III C2 after surgery due to lymph node metastasis (LNM) based on the 2018 FIGO staging criteria. Of these, 60 (19.3%) experienced relapse. The 1-, 3-, and 5-year RFS rates were 93.9%, 82.7%, and 79.3%, respectively. Multivariate analysis revealed that the number of positive lymph nodes (LNs), tumor diameter (TD) > 4 cm, and parametrial invasion (PI) were associated with recurrence. The nomogram indicated their predictive value for 3-year and 5-year RFS. Notably, the 5-year recurrence rate (RR) increased by 30.2% in patients with LNM, particularly those with ≥ 3 positive LNs (45.5%). Patients with stage III C2 exhibited a significantly higher RR than those with IIIC1 (56.5% vs. 24.3%, *p* < 0.001). The 5-year RFS for patients with TD > 4 cm was 65.8%, significantly lower than for those with TD ≤ 4 cm (88.2%). Subgroup analysis revealed higher 5-year RRs in patients with stage III C2 than that in patients with III-C1 (56.5% vs. 24.3%, *p* < 0.001), demonstrating a significant difference in the RFS survival curve.

**Conclusion:**

RR in patients with clinical ESCC after hysterectomy followed by adjuvant radiotherapy is correlated with the number of positive LNs, TD > 4 cm, and PI. Emphasis should be placed on the common high-risk factor of LNM association with recurrence after radical hysterectomy in ESCC.

## Introduction

Cervical cancer (CC) ranks as the fourth most frequently diagnosed cancer and the fourth leading cause of cancer-related mortality [1]. Over the past decade, CC mortality has seen a gradual increase in transitioning countries such as China, primarily due to inadequate human papillomavirus (HPV) vaccination and screening strategies [2, 3]. While both surgery and radiotherapy are viable options for clinical early-stage cervical cancer (ESCC), surgery is more commonly employed in developing countries. However, a subset of patients experiences relapse after several years, with a limited survival time post-recurrence, often not surpassing 12 months [4]. Postoperative risk factors have been traditionally categorized as high and intermediate risk; however, the specific impact of each on prognosis remains underexplored.

Many large-scale clinical trials have predominantly utilized the older The International Federation of Gynecology and Obstetrics (FIGO) stage (2009 edition) or subsequent staging standards. FIGO has introduced a new staging system in 2018. However, the extent to which this updated staging system can guide clinical practice and accurately predict prognosis has not been thoroughly investigated. Moreover, there is a paucity of research data concerning the prognosis of patients initially diagnosed with ESCC who undergo surgery when lymph node metastasis (LNM) is identified postoperatively and during adjuvant therapy. This study aims to analyze recurrence-related factors in patients with ESCC following hysterectomy and adjuvant radiotherapy, also seeking to validate the revised 2018 FIGO staging system for CC.

## Materials and methods

### Study design and patients

This retrospective study, conducted at Zhongshan Hospital affiliated with Fudan University, screened patients diagnosed with ESCC (e.g., I B1, I B2, II A1, and II A2) by the FIGO 2009 staging system between January 2012 and December 2019. Patients underwent radical hysterectomy, lymphadenectomy, and adjuvant radiotherapy or chemoradiothrapy. Retrospective restaging was performed based on surgical pathological characteristics using the FIGO 2018 staging system. All patients received postoperative adjuvant radiotherapy at the Department of Radiation Oncology. The study received approval from the Ethics Committee of Zhongshan Hospital, affiliated with Fudan University, and obtained written informed consent from each patient (B2021-814R).

Inclusion criteria: All adult patients who underwent radical hysterectomy and pelvic lymph node (LN) dissection, employing surgical or laparoscopic approaches, were included. Surgical stages ranged from I B1–I B3, II A1–A2, II B to III C1-2, without evidence of distant metastasis on preoperative imaging (magnetic resonance imaging [MRI] and/or computed tomography [CT] and/or positron emission tomography [PET]/CT). Exclusion criteria encompassed patients with malfunctioning vital organs (heart, liver, bone marrow, and kidney), a medical history of additional malignant tumors, or prior radiotherapy or chemotherapy. Patients with intermediate risk factors (IRFs), as per Sedlis criteria based on GOG92, necessitated adjuvant radiation [5]. Patients with adenocarcinoma were referred to the KGOG Study [6], and those with two or more risk factors will received adjuvant radiotherapy. High-risk factors (HRFs) included parametrial, LN, or vaginal cut margin involvement, mandating concurrent chemoradiotherapy (CCRT) based on GOG109 results [7].

### Gynecological surgical methods

All eligible patients underwent radical hysterectomy combined with pelvic LN dissection ± para-aortic LN biopsy/dissection using either a surgical or laparoscopic approach. The laparoscopic approach was discontinued at our center following the publication of the Laparoscopic Approach to Cervical Cancer (LACC) results in 2018 ((8–9)). Radical hysterectomy involved the removal of the uterus, parametrium, paravaginal tissues, upper third of the vagina, and uterosacral ligament. The ureter was dissected from its entry into the broad ligament to its reach to the bladder and laterally from its attachment to the cardinal ligament.

### Adjuvant radiotherapy

Adjuvant radiotherapy generally employed intensity-modulated radiotherapy or three-dimensional conformal radiotherapy (6-MV photon beam). Based on the Radiation Therapy Oncology Group target delineation guidelines, the clinical target volume (CTV) primarily included common, external, and internal iliac LN regions, presacral LN region, and the upper 3.0 cm of the vagina and paravaginal soft tissue lateral to the vagina [10]. The planning target volume (PTV) was defined by expanding an edge of 0.5–1.0 cm to the CTV. The prescribed dose was 45.0–50.4 Gy/1.8 Gy, five times per week. The minimum and maximum acceptable PTV dose were 95% and 110% of the prescribed dose (median 45 Gy), respectively. Most patients received a radiation field of the pelvic cavity. Patients with a positive vaginal margin received intracavitary brachytherapy with a dose at point A of 30 Gy ± 10%, 5–6 Gy per fraction, once or twice a week for 4–6 fractions in total. Patients with common iliac or para-aortic lymph node metastases received para-aortic extension fields (simultaneously integrated or sequential). All treatments commenced 2–4 weeks following surgery and were completed within 6–8 weeks. The limits of the organ-at-risk were as follows: The maximum dose of the spinal cord was less than 45 Gy. small intestine V_40_ < 30%,V_30_ < 40%; the average dose of bilateral femoral head < 30 Gy; bladder V_45_ < 40%;rectumV_40_ < 40%; left and right kidney V_20_ < 33%.

### Adjuvant chemotherapy regimen

The decision between CCRT or radiotherapy was determined by the treatment team, considering patients’ risk factors, performance scores, and comorbid conditions. Patients with HRFs received cisplatin-based combination chemotherapy once a week (30–40 mg/m^2^) for 5 weeks. The decision to administer synchronous chemotherapy to patients with IRFs was collaboratively taken following discussions among the doctor, patient, and their families. A subset of patients received postoperative adjuvant chemotherapy with paclitaxel (135–175 mg/m^2^) combined with cisplatin (60–75 mg/m^2^) or carboplatin (area under the curve [AUC] = 5) every 3 weeks following radiotherapy for 4–6 cycles. The treatment team considered factors such as patients’ age, HRFs, performance scores, and comorbid conditions for making this decision. Due to the side effects of bone marrow suppression, most patients completed 5 weeks of synchronous chemotherapy, whereas patients at high risk received 4 cycles of postoperative adjuvant chemotherapy.

### Follow-up plan

Patients underwent regular follow-ups with a frequency of every 3 months during the first 2 years, every 6 months in 3–5, and annually after 5 years. The follow-ups included medical examinations, outpatient colposcopy, ultrasonography, CT scans, MRI, PET/CT, and serum tumor biomarker detection (usually adopted in combination rather than all). Recurrence is defined as evidence of tumor relapse or metastasis detected in any medical examination after a minimum of 3 months following the completion of the operation, including local recurrence (within the radiation field, e.g., pelvic cavity or irradiated para-aortic extension fields) and distant metastasis (LNM outside the radiation field area, lung metastasis, bone metastasis, and other organ metastasis). Survival calculations were performed post-surgery.

### Statistical analysis

IBM SPSS (version 22.0, Chicago, USA) and R software (Version 4.1.2) were utilized for statistical analyses. Descriptive statistics summarized the frequency of clinical-pathological factors (e.g., age, FIGO Stage, parametrial invasion (PI), surgical margin, number of LNM, depth of stromal invasion, tumor diameter (TD), lymphovascular space invasion (LVSI), and histological type). The Pearson χ2 test or Fisher’s exact test determined the difference in 5-year recurrence-free survival (RFS) rates between subgroups. The Cox proportional hazards model was employed to establish the relationship between each clinical/demographic factor and RFS. Factors identified by univariable analyses (*p* < 0.1) (FIGO stage, PI, surgical margin, TD, number of of LN+) were subjected to multivariable analyses to identify significant independent factors. The R software was used to draw a nomogram, assessing the value of prognosis-related factors in predicting outcomes. The accuracy of the prediction model was evaluated using an internal calibration curve. Survival curves and the number of at-risk patients were generated and calculated using R software. The Kaplan–Meier method estimated the median overall survival (OS), and the log-rank test compared the association of OS and RFS with relevant clinicopathological factors between subgroups. The significance level was set at 0.001 due to multiple testing, adhering to the Bonferroni adjustment. All *p*-values were two-sided, and a *p*-value less than 0.05 was considered statistically significant.

## Results

### Patient characteristics

A total of 504 cases of ESCC were screened from the radiotherapy database of Zhongshan Hospital between January 2012 and December 2019, meeting the inclusion criteria. Among these, 105 patients with radical and palliative radiotherapies, 59 patients with incomplete surgical data, and 30 patients lacking follow-up data were excluded. Ultimately, 310 patients were enrolled, with a mean age of 50.9 years. Of these, 60 (19.4%) experienced recurrence, and 52 (16.8%) succumbed to the disease. The median prescribed dose was 45.0 Gy (45.0–50.4 Gy), and the median follow-up time was 46 months (5.7–119.4 months). CCRT was administered to 185 (59.7%) patients, and 113 (36.5%) received adjuvant chemotherapy. The 1-, 3-, and 5-year RFS rates for patients with ESCC were 93.9%, 82.7%, and 79.3%, respectively. The median RFS was not determined. Based on the 2018 FIGO staging system, 85, 99, and 126 patients were categorized as stages I, II, and III C, respectively, with corresponding 5-year RFS rates of 92.5%, 85.1%, and 71.9%. Among them, 100 (32.3%) patients were restaged as III C1, and 26 (8.4%) as III C2. Additionally, 26 patients initially classified as stage I B1 by the FIGO 2009 staging were reclassified post-surgery, with 18 showing no disease progression, Among these patients, 1 was reclassified as stage I B2, 1 as IB3, 1 as II B, and 5 as III C1. According to the new staging system, the 1-, 3-, and 5-year RFS rates for stage IB1 were 100%, 94.4%, and 94.4%, respectively, and the 1-, 3-, and 5-year RFS rates for stage IB2 were 97.4%, 91.8%, and 91.8%, respectively. No statistical difference was observed between the I B1 and I B2 groups in terms of RFS or OS. Pathological features, 5-year recurrence rate (RR), and 5-year RFS are summarized in Table 1.

**Table 1:** Patient characteristics and analyses based on recurrence-free survival status

Parameter	n (%^a^)	Total recurrence	5-y RR	5-y RFS	*p*-value
		n(%)	%	n (%^b^)	
Age					
≤ 40	45 (14.5)	11	24.4	34(72.6)	0.431
40–60	199(64.2)	37	16.6	166(81.2)	
> 60	66 (21.3)	12	18.2	54(77.3)	
FIGO clinical stage (2009)					0.002
I B1	26	3	11.5	23(88.1%)	
I B2	109 (31.9)	15	13.7	94(84.6%)	
II A1	91 (33.1)	18	18.7	74(79.7%)	
II A2	84(7.6)	24	27.4	61(69.3%)	
FIGO surgical restage (2018)					< 0.001*
I	83(26.8)	6	7.2	77(92.5)	
I B1	17(5.5%)	1	5.9	16(94.1%)	
I B2	40(12.9%)	3	7.5	37(92.0%)	
I B3	26(8.4%)	2	7.7	24(92.3%)	
II	101 (32.6)	16	14.1	85(85.1)	
II A1	50(16.1%)	7	12	44(86.9%)	
II A2	29(9.4%)	3	31	27(91.2)	
II B	22(7.1%)	6	27.3	16(72.7%)	
III C1	104 (33.5)	26	25	78(75.7%)	
III C2	22(7.1)	12	54.5	10(42.4%)	
Risk factors					< 0.001
high-risk	145	44	34.5	95(65.5)	
intermediate-risk	165	16	9.1	150(90.9)	
Parametrial invasion					< 0.001
Pos	28(9.0)	19	62.9	9(32.1)	
Neg	282(91.0)	41	16.7	234(83.3)	
Positive vaginal margin					0.006
Pos	17(5.5)	8	47.1	9(52.9)	
Neg	293(94.5)	52	19.1	237(80.9)	
No. of LN+					< 0.001
≥ 3	44(14.2)	20	52.3	21(47.7)	
1–2	82(26.5)	18	24.4	62(75.6)	
0	184(59.3)	22	11.4	163(88.6)	
Depth of stromal invasion					0.022
Deep 1/3	204(65.8)	47	25	153(75)	
Middle 1/3	77(24.8)	11	14.3	66(85.7)	
Superficial 1/3	29(9.4)	2	6.9	27(93.1)	
Tumor diameter					< 0.001
> 4 cm	123(39.7)	40	34.2	81(65.8)	
≤ 4 cm	187(60.3)	20	11.8	165(88.2)	
LVSI					0.029
Pos	203(65.5)	44	24.6	153(75.4)	
Neg	107(34.5)	16	14	92(86.0)	
Nerve invasion					0.161
Pos	41(13.2)	10	29.3	29(70.7)	
Neg	269(86.8)	50	19.7	216(80.3)	
Vaginal invasion					
Yes	110(35.5)	26	23.6	83(75.5)	0.171
No	200(64.5)	34	17	164(82)	
Histological type					0.535
SCC	265 (85.5)	49	20.4	211(79.6)	
Non-SCC	45 (14.5)	11	24.5	34(75.5)	

^a^ percentage in the whole patients, ^b^ percentage in each subgroup. *compared RR difference among stage I, II, IIIC1 and IIIC2

Abbreviation: RR: recurrence rate; RFS: recurrence-free survival; FIGO, International Federation of Gynecology and Obstetrics; LVSI, lymphovascular space involvement; SCC, squamous cell carcinoma. *p*-values were compared RR difference among subgroups of clinical/demographic factor.

Bold font: *p*-value < 0.05

### Recurrence-related pathological factors

**Fig. 1:**
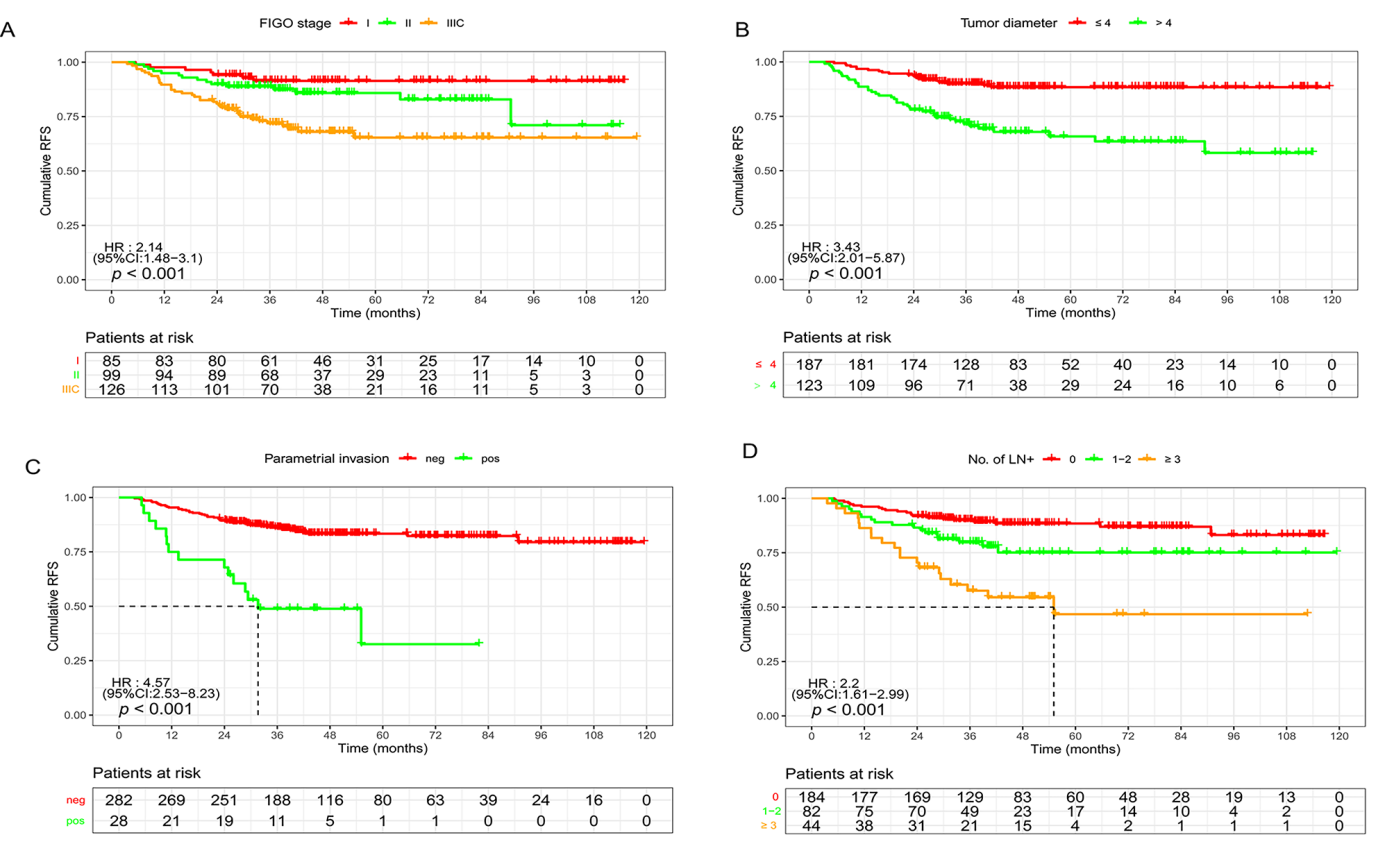
Stratified analyses of recurrence-free survival (RFS) based on risk factors. (**A**) Survival analyses of patients based on the FIGO stage (I, II, and III C: 92.5%, 85.1%, and 71.9%, respectively). (**B**) Five-year RFS rates of patients based on tumor diameter (> 4 cm vs. ≤4 cm; 65.8% vs. 88.2%, respectively). (**C**) Five-year RFS rates of patients with ESCC stratified by parametrial invasion (pos. vs. neg.; 32.1% vs. 83.3%, respectively). (**D**) Five-year RFS stratified by number of positive lymph nodes (0, 1–2, vs. ≥3, 88.6%, 75.6%, vs. 47.7%, respectively)

Univariate analysis revealed associations between recurrence and the number of positive LNs, positive vaginal margin, PI, depth of invasion, and TD Multivariate analysis identified the number of positive LNs, PI, and TD as independent factors affecting RR (Table 2). Survival analysis demonstrated that the number of positive LNs, PI, and TD were independent prognostic factors for RFS in the entire cohort. Postoperative clinicopathological analysis revealed that the 5-year RFS of patients with TD > 4 cm was significantly lower than patients with TD ≤ 4 cm (65.8% vs. 88.2%, *p* < 0.001). The 5-year RFS rate of patients without PI was lower than that of those with this factor (32.1% vs. 83.3%, *p* < 0.001). The 5-year RR of patients with positive LNM was significantly higher than those without LNM (30.2% vs. 11.4%, *p* < 0.001). Notably, in patients with 3 or more positive LNs, the RR was 45.5%. These results are summarized in Tables 1 and 3 and visually depicted in Fig. 1.

**Table 2:** Univariate and multivariate analyses of risk factors for recurrence-free survival

Parameter	univariate					multivariate	
	HR	95.0% CI	*p-*value	HR		95.0% CI	*p-*value
Age				0.621			
≤ 40	1.38	0.61–3.13		0.442			
40–60	0.99	0.52–1.91		0.986			
> 60	1[Reference]						
Vaginal invasion							
Yes	1.48	0.89–2.47		0.134			
No	1[Reference]						
FIGO Stage				**< 0.001**			
I	0.27	0.12–0.58		**< 0.001**			
II	0.46	0.25–0.84		**0.012**			
III C	1[Reference]						
Parametrial invasion							
Pos	4.62	2.56–8.33		**< 0.001**	3.19	1.74–5.85	**< 0.001**
Neg	1[Reference]						
Surgical margin							
Pos	3.23	1.53–6.82		**0.002**			
Neg	1[Reference]						
No. of LN+				**< 0.001**	1.99	1.46–2.71	**< 0.001**
≥ 3	4.32	2.35–7.96		**< 0.001**	3.96	2.12–7.36	**< 0.001**
1–2	1.95	1.05–3.62		**0.034**	1.98	1.06–3.72	**0.032**
0	1[Reference]				1[Reference]		
Depth of stromal invasion				0.081			
Deep 1/3	2.29	0.97–5.37		0.058			
Middle 1/3	1.37	0.50–3.76		0.544			
Superficial 1/3	1[Reference]						
Tumor diameter							
> 4	3.42	2.00-5.86		**< 0.001**	3.24	1.89–5.54	**< 0.001**
≤ 4	1[Reference]						
LVSI							
Pos	1.47	0.85–2.54		0.169			
Neg	1[Reference]						
Nerve invasion							
Pos	1.44	0.73–2.85		0.291			
Neg	1[Reference]						
Histological type							
SCC	0.62	0.33–1.18		0.145			
Non-SCC	1[Reference]						

Abbreviation: HR, hazard ratio; CI: confidence interval; FIGO, International Federation of Gynecology and Obstetrics; LVSI, lymphovascular space involvement; SCC, squamous cell carcinoma. Bold font: *p-*value < 0.05

**Table 3:** Univariate and multivariate analyses of risk factors for recurrence-free survival in LNM group

Parameter	No. of patients	5-y RR	univariate			multivariate		
		n(%)	HR	95.0% CI	*p* value	HR	95.0% CI	*p* value
Age					0.848			
≤ 40	26(20.6)	8(30.8)	0.83	0.25–2.76	0.765			
40–60	77(61.1)	22(28.6)	0.75	0.28–2.02	0.569			
> 60	23(18.3)	8(34.8)	1[Reference]					
FIGO stage								
III C2	23(18.3)	13(56.5)	4.06	1.59–10.38	**0.003**			
III C1	103(81.7)	25(24.3)	1[Reference]					
Parametrial invasion								
Pos	17(13.5)	12(70.6)	7.66	2.47–23.78	**< 0.001**	3.51	1.76-7.00	**< 0.001**
Neg	109(86.5)	26(23.9)	1[Reference]					
Positive vaginal margin								
Pos	8(6.3)	5(62.5)	4.29	0.97–18.99	**0.055**			
Neg	118(93.7)	33(28.0)	1[Reference]					
No. of LN+					**0.007**			
≥ 3	44(34.9)	20(45.5)	2.96	1.34–6.54		1.94	1.02–3.69	**0.043**
1–2	82(65.1)	18(22.0)	1[Reference]					
Depth of stromal invasion					0.053			
Deep 1/3	83(65.9)	31(37.3)	10.14	1.29–79.93	**0.028**			
Middle 1/3	25(19.8)	6(24.0)	5.37	0.59–49.22	0.137			
Superficial 1/3	18(14.3)	1(5.6)	1[Reference]					
Tumor diameter								
> 4	53(42.1)	25(56.6)	4.12	1.84–9.23	**0.001**	2.95	1.51–5.77	**0.002**
≤ 4	73(57.9)	13(19.2)	1[Reference]					
LVSI								
Pos	93(73.8)	31(39.8)	1.86	0.73–4.75	0.196			
Neg	33(26.2)	7(21.2)	1[Reference]					
Nerve invasion								
Pos	17(13.5)	7(41.2)	1.76	0.62–5.04	0.291			
Neg	109(86.5)	31(28.4)	1[Reference]					
Histological type								
SCC	106(84.1)	31(29.2)	1.30	0.48–3.58	0.608			
Non-SCC	20(15.9)	7(35.0)	1[Reference]					

Abbreviation: HR, hazard ratio; CI: confidence interval; FIGO, International Federation of Gynecology and Obstetrics; LVSI, lymphovascular space involvement; SCC,squamous cell carcinoma. Bold font: *p* value < 0.05

### Prediction of the impact of related pathological factors on RFS using the nomogram

**Fig. 2:**
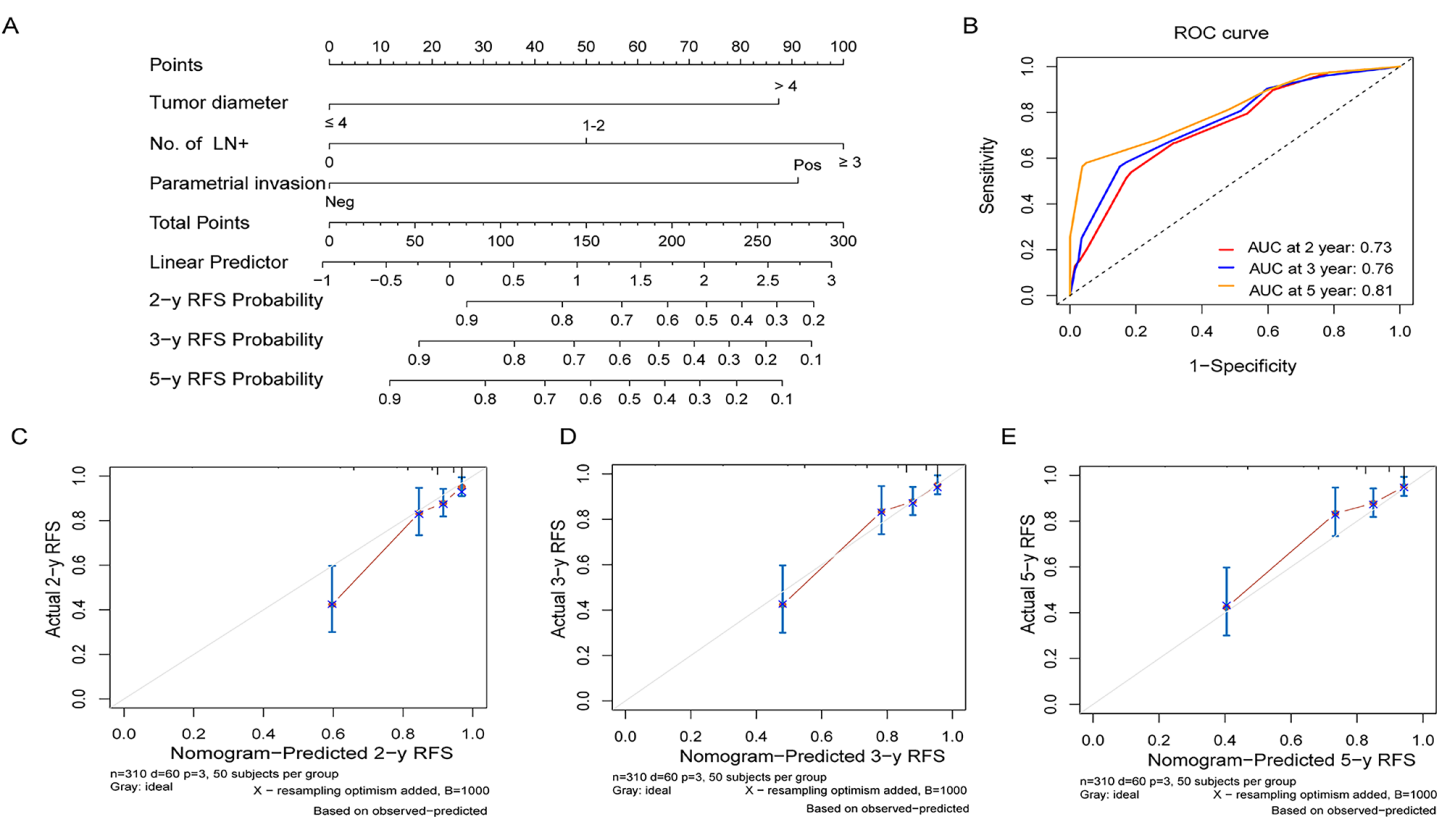
Nomogram and calibration curves of patients with ESCC. (**A**) A nomogram for the prediction of 2-, 3- and 5-year RFS in patients with ESCC. (**B**) Receiver operating characteristic curve for the prediction of 2-, 3- and 5-year RFS. Calibration curves of the nomogram prediction of (**C**) 2-, (**D**) 3-, and (**E**) 5-year survival of patients with ESCC

Following the results of multivariate analysis, a nomogram was developed to predict the RFSof patients with ESCC (Fig. 2A). The summation of individual prognostic factor scores on the nomogram provided the patient’s total score, corresponding to their risk ratio for 2-, 3-, and 5-year RFS rates. The C-index of the prediction model was 0.76, indicating good predictive efficiency. To verify accuracy, a receiver operating characteristic curve was plotted, revealing an AUC at 2-, 3-, and 5-year timepoints of 0.73, 0.76, and 0.81, respectively (Fig. 2B). Calibration curves of nomogram predictions at 2-, 3-, and 5-year intervals, estimated by a 4-point scale based on 1000 guide charts, demonstrated good consistency between actual and predicted RFS (Fig. 2C, D, and E).

### Subgroup analyses

**Fig. 3:**
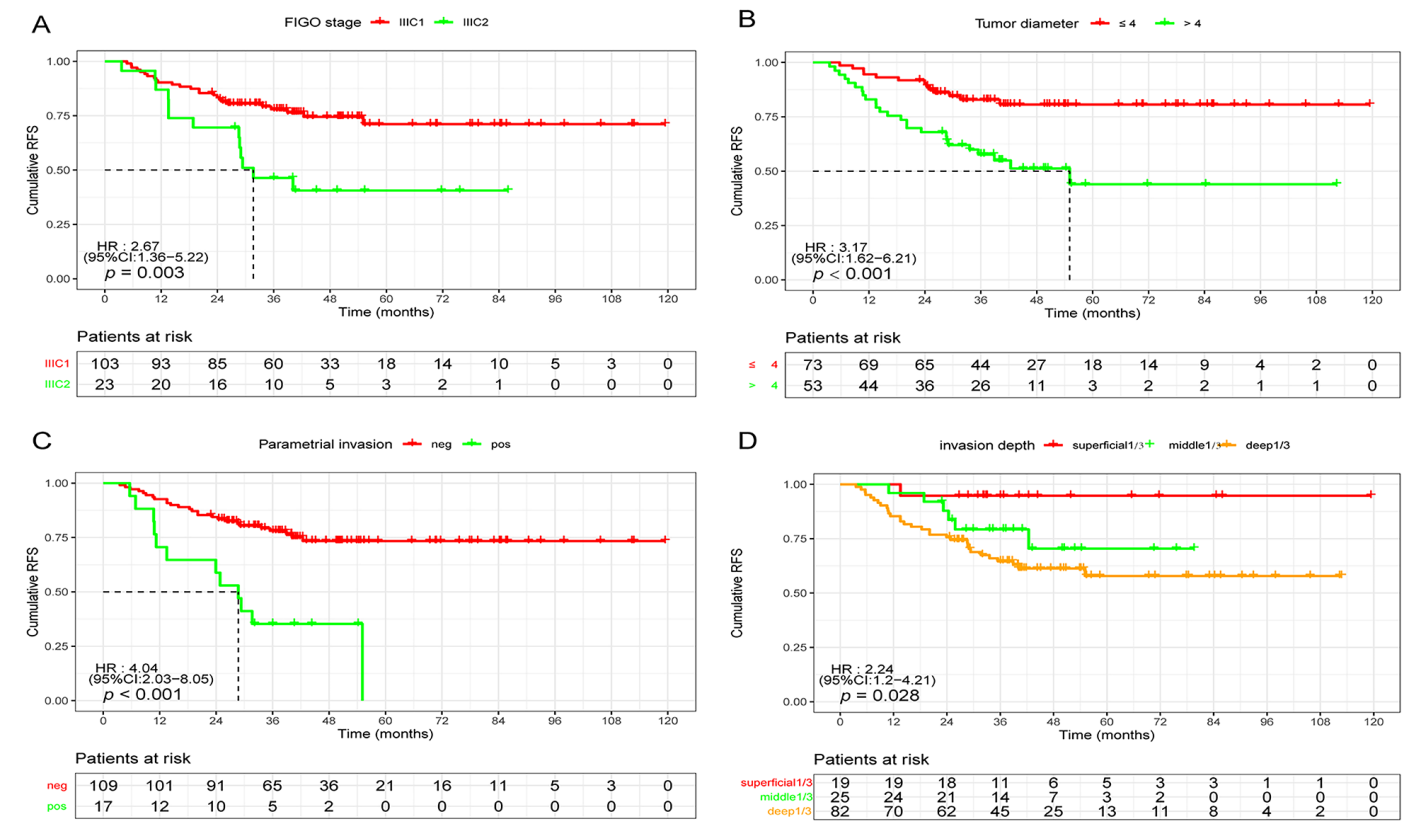
RFS stratified analyses based on risk factors in patients with LNM. (**A**) Five-year RFS analyses of patients based on tumor location (III C1 vs. III C2; 75.7% vs. 42.4%, respectively). (**B**) Five-year RFS analyses of patients with ESCC based on tumor diameter (> 4 cm vs. ≤4 cm; 43.4% vs. 80.8%, respectively). (**C**) Five-year RFS stratified by parametrial invasion (pos. vs. neg.; 29.4% vs. 76.1%). (**D**) Five-year RFS analyses of patients based on the depth of invasion (deep1/3, middle 1/3 vs. superficial 1/3, 62.7%, 76% vs.94.4%)

Among the patients, 145 (46.7%) exhibited HRFs, and 53.3% exhibited IRFs. Of those with HRFs, 126 (86.9%) exhibited LN involvement, 28 (19.3%) exhibited PI, and 17 (11.7%) demonstrated positive vaginal margins. Some patients exhibited one or more HRFs. In patients with LNM, multivariate analyses revealed that the number of positive LNs, TD > 4 cm, and PI were independent recurrence-related factors. Subgroup analysis revealed that the 5-year RRs of patients with stage III C2 were higher than those with III C1 (56.5% vs. 24.3%, *p* = 0.003), showing a significant difference in the RFS survival curve. Patients with three or more positive LNs exhibited a higher RR than those with 1–2 LNs (45.5% vs. 22.0%, *p* < 0.001). Moreover, a crucial recurrence-related factor was the depth of PI, with a higher 5-year RR in patients with PI than in those without PI (70.6% vs. 23.9%, *p* < 0.001) in the LNM subgroup (Table 3; Fig. 3).

### Recurrence pattern

Out of the total patients, 60 (19.3%) experienced recurrence, with the majority presenting clinical-pathological factors of TD > 4 cm (66.7%) and deeper 1/3 invasion (78.3%). Among them, 22 (36.7%) had local recurrence, whereas 38 (63.3%) experienced distant metastasis, predominantly to the lungs, as well as bone and distant LN metastases outside the radiation field. The RR of the LNM group was significantly higher than that of the non-LNM group (34.1% vs. 11.4%; *p* < 0.001). However, further analysis revealed that the failure patterns of local recurrence or distant metastasis in the two groups were similar, with no significant differences (Table 4).

**Table 4:** Recurrence patterns in LNM or non-LNM patients

Recurrence patterns	Non-LNM	LNM	Total	*p* value
	*n* = 184	*n* = 126	*N* = 310	
No relapse	162(89.1)	88 (72.2)	255 (82.3)	< 0.001
Relapse*	22(11.9)	38(30.1)	55 (17.7)	
Reginal	9 (4.9)	14 (11.1)	23 (7.4)	0.683
Distant	15 (8.7)	29(16.7)	32(10.3)	

*Some patients had more than one region of relapse

LNM: lymph node metastasis

## Discussion

This study encompasses a comprehensive data collection of 310 ESCC cases from real-world scenarios after hysterectomy followed by adjuvant radiotherapy or chemoradiotherapy in a single-institution cohort. The data is more authentic and heterogeneous, aligning more closely with actual clinical practice. While the 2009 FIGO staging is simpler for surgical physicians, its prognostic significance is deemed insufficient. In this study, 126 patients with ESCC were reclassified to stage III C1 or III C2 post-surgery due to LNM based on the 2018 FIGO staging criteria. They exhibited a poorer 5-year RFS than those whose disease staging had not changed (65.9% vs. 88.6%), even after receiving CCRT. Similar findings in another study indicated a reduction in the 5-year RFS rate from 88 to 57% in the LNM group [11], confirming the efficacy of the the 2018 staging system in guiding the prognosis of patients with LN positivity.

The KROG1303 study constructed a nomogram incorporating parameters such as age, number of pelvic LNMs, PI, LVSI, and the use of CCRT to predict 5-year OS. The risk of death increased with the number of pelvic LNMs [12]. Another multicenter retrospective study, including 249 IB to II A patients, identified the number of LNMs as the best prognostic variable related to LN status, and patients with LNM > 3 had a high risk of recurrence even with postoperative chemoradiotherapy [13]. Our findings are consistent with these results, with multivariate analysis indicating that the number of positive LNs was an independent factor affecting recurrence and an independent prognostic factor of RFS. Patients with 3 or more positive LNs had a higher RR than those with 1–2 LNs (45.5% vs. 22.0%). However, besides the number of LNMs, factors such as the location, distribution, proportion, and size of LNMs may also be relevant to prognosis, warranting further research and exploration.

Despite the recommendation for postoperative CCRT for all patients with LNM in this study, the follow-up data revealed that patients with LNM still exhibited the worst prognosis. This suggests that LNM may differ from other HRFs such as positive vaginal margins and PI, whose risks might be mitigated by brachytherapy. LNM is viewed as a systemic disease with microscopic tumor spread, and systemic treatment may be more effective than local treatment for patients with LNM. This hypothesis requires further investigation.

LNM is a crucial factor in treatment decisions and prognosis prediction. Preoperative evaluation of LNM is therefore pivotal in clinical practice. After the revision of staging in 2018, patients with stage III C are more likely to receive CCRT-based treatment. Determining LNM accurately before surgery is challenging, and literature indicates the importance of PET/CT in identifying low-risk patients [14]. While PET/CT is not covered by medical insurance in China and poses financial challenges for some patients, the data and findings highlight the significance of performing PET/CT in patients with ESCC preparing for surgery, not only those undergoing definitive radiotherapy.

Despite tumor size being considered an IRF, our research findings reveal that tumor size is an independent factor affecting recurrence, as crucial as the classic HRFs of PI and LNM. This contrasts with the traditional view that smaller tumors are less dangerous in terms of recurrence and metastasis. A study supporting our findings demonstrated that, for patients with stage III C1, the prognosis closely correlated with the extent of tumor invasion, with a wider scope of invasion leading to a worse prognosis. Another study showed that the 5-year survival rates of T1, T2, and T3 were 74.8%, 54.7%, and 39.4%, respectively, among patients with stage III C1 [15]. In our study, we observed that in the LNM-positive subgroup, patients with TD > 4 cm and deeper PI had a poorer prognosis. These findings cast doubt on the rationality of dividing postoperative recurrence risk factors into HRF and IRF categories. Currently, the NCCN guidelines recommend that patients with ESGC with IRFs should undergo radiotherapy ± chemotherapy. The evidence level of concurrent radiotherapy and chemotherapy for moderate risk factors is category II B from a single cohort. Our findings indicate that for patients with large TD, the recurrence probability is very high, and CCRT should be strongly recommended for such patients in clinical practice.

No statistical difference was found between the IB1 and IB2 groups in terms of RFS or OS in this study, possibly due to the small sample size. In contrast, another study comparing the old system with the 2018 FIGO new staging system in a validation study demonstrated that the 5-year disease-free survival of the old I B1 (5 mm to 4 cm) was 90.0%, whereas that of the new I B2 (2–4 cm) was 78.6%. This new classification was deemed more effective in guiding the prognosis of patients [16]. It is worth noting that our study covers all pathological classifications, which could significantly impact statistical results. For instance, a patient diagnosed with gastric-type endocervical adenocarcinoma with stage I B1 experienced rapid disease progression and recurrence 29.8 months following surgery. The survival time of this patient could significantly affect that of the whole IB1 group due to the small sample size. In conclusion, we were unable to complete the validation of the effect of dividing old I B1 into I B1 and I B2 stages according to the boundary value of 2 cm in diameter in the 2018 FIGO stage in this study.

Despite the robustness of our study, several limitations need to be acknowledged. The retrospective design and the modest number of patients might compromise statistical accuracy. The absence of a significant difference between the IB1 and IB2 groups in terms of RFS or OS may be attributed to the small number of cases, emphasizing the need for caution in the interpretation of these results. The inclusion of all pathological classifications in our study could significantly impact statistical outcomes. Furthermore, the lack of detailed classification regarding the characteristics of the location, number, and size of metastatic lymph nodes could potentially influence prognostic assessments and should be addressed in future research. Additionally, the study did not include data on the status of HPV infection, which is recognized as a significant risk factor for recurrence in patients undergoing conization for high-grade cervical lesions [17]. Future studies should consider incorporating this crucial aspect for a more comprehensive analysis. Moreover, the suspension of laparoscopic surgery since November 2018 in our hospital raises questions, although new research results seem to be inconsistent with LACC trial in low-risk patients [18]. Thus, high-level evidence from phase III studies are urgently required on how to select between these two approaches.

## Conclusion

The challenges posed by the recurrence and metastasis of EGCC persist as clinical complexities, and ongoing debates surround the primary determinants of prognosis. In this retrospective study, we meticulously examined 310 patients diagnosed with ESCC, aiming to understand the pivotal factors influencing recurrence. Our findings reveal that the RR in patients with clinical ESCC post-hysterectomy, followed by adjuvant radiotherapy, is associated with specific factors—namely, the number of positive LNs, TD exceeding 4 cm, and PI. Of utmost significance is the imperative to direct heightened attention toward LNM and TDs. Patients manifesting these factors may necessitate more aggressive intervention strategies and tailored treatment approaches.

## Data Availability

The datasets used for analysis in the current study are available from the corresponding author on reasonable request.

## Abbreviations

HPV: Human papillomavirus
ESCC: Early-stage cervical cancer
FIGO: Federation International of Gynecology and Obstetrics
RFS: Recurrence-free survival
LNM: Lymph node metastasis
TD: Tumor diameter
PI: Parametrial invasion
RR: Recurrence rate
LN: Lymph node
CCRT: Concurrent chemoradiation
HRF: High risk factors
IRF: Intermediate risk factors
